# Deep-learning framework and computer assisted fatty infiltration analysis for the supraspinatus muscle in MRI

**DOI:** 10.1038/s41598-021-93026-w

**Published:** 2021-07-23

**Authors:** Kyunghan Ro, Joo Young Kim, Heeseol Park, Baek Hwan Cho, In Young Kim, Seung Bo Shim, In Young Choi, Jae Chul Yoo

**Affiliations:** 1 Gangnambon Research Institute, Gangnambon Orthopaedic Cinic, Seoul, Republic of Korea; 2 Department of Biomedical Engineering, Hanyang University, Seoul, Republic of Korea; 3 Department of Orthopaedic Surgery, Samsung Medical Center, Sungkyunkwan University School of Medicine, Seoul, Republic of Korea; 4 Medical AI Research Center, Samsung Medical Center, Sungkyunkwan University School of Medicine, Seoul, Republic of Korea; 5 Department of Orthopaedic Surgery, Yonsei Thebaro Hospital, Seoul, Republic of Korea; 6 Department of Radiology, Korea University Ansan Hospital, Korea University, Ansan-si, Gyeonggi-do Republic of Korea; 7 Department of Medical Device Management and Research, SAIHST, Samsung Medical Center, Sungkyunkwan University School of Medicine, Seoul, Republic of Korea

**Keywords:** Clinical trial design, Musculoskeletal system

## Abstract

Occupation ratio and fatty infiltration are important parameters for evaluating patients with rotator cuff tears. We analyzed the occupation ratio using a deep-learning framework and studied the fatty infiltration of the supraspinatus muscle using an automated region-based Otsu thresholding technique. To calculate the amount of fatty infiltration of the supraspinatus muscle using an automated region-based Otsu thresholding technique. The mean Dice similarity coefficient, accuracy, sensitivity, specificity, and relative area difference for the segmented lesion, measuring the similarity of clinician assessment and that of a deep neural network, were 0.97, 99.84, 96.89, 99.92, and 0.07, respectively, for the supraspinatus fossa and 0.94, 99.89, 93.34, 99.95, and 2.03, respectively, for the supraspinatus muscle. The fatty infiltration measure using the Otsu thresholding method significantly differed among the Goutallier grades (Grade 0; 0.06, Grade 1; 4.68, Grade 2; 20.10, Grade 3; 42.86, Grade 4; 55.79, *p* < 0.0001). The occupation ratio and fatty infiltration using Otsu thresholding demonstrated a moderate negative correlation (*ρ* = − 0.75, *p* < 0.0001). This study included 240 randomly selected patients who underwent shoulder magnetic resonance imaging (MRI) from January 2015 to December 2016. We used a fully convolutional deep-learning algorithm to quantitatively detect the fossa and muscle regions by measuring the occupation ratio of the supraspinatus muscle. Fatty infiltration was objectively evaluated using the Otsu thresholding method. The proposed convolutional neural network exhibited fast and accurate segmentation of the supraspinatus muscle and fossa from shoulder MRI, allowing automatic calculation of the occupation ratio. Quantitative evaluation using a modified Otsu thresholding method can be used to calculate the proportion of fatty infiltration in the supraspinatus muscle. We expect that this will improve the efficiency and objectivity of diagnoses by quantifying the index used for shoulder MRI.

## Introduction

With the advancements in computer-aided diagnoses, aided by deep-learning frameworks and convolutional neural networks (CNNs), several methods have been made to analyze medical images using artificial intelligence^1–3^.

Atrophy and fatty infiltration of the supraspinatus muscle, as observed in magnetic resonance imaging (MRI), can reveal the severity of a rotator-cuff tear^4^. Atrophy of rotator-cuff muscles is one of the most important prognostic factors for anatomical and functional results following surgical repair^5^. Furthermore, fatty infiltration of the supraspinatus muscle not only aggravates functional outcomes, but also increases the re-tear rate after repair^6,7^. However, accurate measurement of these indices often relies on clinicians. This is a time-consuming process, and there have been debates about their accuracy and reliability^8^.

We hypothesize that segmentation of the supraspinatus muscle and fossa via deep learning will achieve excellent results compared with human observations and measurements. However, the quantitative value of fatty infiltration of the supraspinatus muscle using computer-assisted analysis might not show significant differences between Goutallier grades 0 and 1 and grades 3 and 4. This study aimed to analyze the occupation ratio using a deep-learning framework and to calculate the amount of fatty infiltration of the supraspinatus muscle using an automated region-based Otsu thresholding technique.

## Results

### Segmentation of the supraspinatus muscle and fossa

The results from the two orthopedic surgeons were in excellent agreement for both the supraspinatus fossa (Dice similarity coefficient [DSC]: 0.88 ± 0.12) and muscle (DSC: 0.91 ± 0.08). The supraspinatus muscle and fossa were segmented using a desktop computer (Intel® Core™ i7-7700 CPU @ 3.60 GHz, 32.0 GB RAM, NVIDIA GeForce GTX 1080 Ti 11 Gbps) in 0.1483 s (148.3369 ms), whereas the segmentation using ITK-SNAP software required more than 5 min for each person. The performance of our proposed models to detect regions of interest compared with clinician findings in terms of DSC, accuracy, sensitivity, specificity, and relative area difference (RAD), are listed in Table 1. The DSC of the supraspinatus fossa was 0.97 ± 0.01 and 0.94 ± 0.05 for the supraspinatus muscle, which reflected excellent agreement. The supraspinatus muscle and fossa showed high accuracy: 99.84 ± 0.08 and 99.89 ± 0.07, respectively. The sensitivity and specificity of the supraspinatus fossa were 96.89 ± 2.20 and 99.92 ± 0.06, respectively. The supraspinatus muscle also showed high sensitivity and specificity: 93.34 ± 7.85 and 99.95 ± 0.03, respectively. The RAD of the supraspinatus muscle was higher than that of the supraspinatus fossa: 0.07% ± 0.01 vs 2.03% ± 9.90.

**Table 1:** Mean dice similarity coefficient, accuracy, sensitivity, specificity, and RAD for segmented areas, comparing clinicians with deep neural network.

Parameter	Supraspinatus fossa	Supraspinatus muscle
DSC	0.97 ± 0.01	0.94 ± 0.05
Accuracy	99.84 ± 0.08	99.89 ± 0.07
Sensitivity	96.89 ± 2.20	93.34 ± 7.85
Specificity	99.92 ± 0.06	99.95 ± 0.03
RAD (%)	0.07 ± 3.71	2.03 ± 9.90

Data are shown as mean ± standard deviation (SD) unless otherwise indicated. DSC = Dice similarity coefficient, RAD = relative area difference.

### Fatty infiltration by Otsu thresholding

Fatty infiltration per Goutallier grade was evaluated for the total shoulder MRI. The interobserver agreement and mean intraobserver agreement of the Goutllier grade between clinicians were 0.78 (good) and 0.87 (excellent) of weighted kappa values, respectively. The intraclass correlation coefficient of the ground truth and prediction was 0.94, indicating excellent agreement. Among the 240 shoulder magnetic resonance (MR) images, 55 had grade 0. Grades 1 and 2 were observed in 75 and 68 images, respectively, which were higher than grades 3 and 4. Quantitative calculation of fatty infiltration via Otsu thresholding was performed. Grade 0 exhibited a value of 0.06 ± 0.14, which was the lowest among the Goutallier grade groups. The fatty infiltration of Goutallier grades 1 and 2 was 4.68 ± 7.21 and 20.10 ± 10.57, respectively. Grade 3 fatty infiltration was 42.86 ± 10.41, and grade 4 exhibited a value of 55.79 ± 10.87. All the differences in fatty infiltration among the Goutallier grade groups were statistically significant (*p* < 0.0001) (Table. 2).

**Table 2:** Analysis of fatty infiltration by OTSU thresholding in each goutallier grade.

Variable		Fatty infiltration			*P-*value
		Mean ± SD	Lower limit	Upper limit	
Goutallier	Grade 0	0.06 ± 0.14	0.00	0.83	< .0001
	Grade 1	4.68 ± 7.21	0.00	29.20	
	Grade 2	20.10 ± 10.57	0.00	40.69	
	Grade 3	42.86 ± 10.41	28.46	62.97	
	Grade 4	55.79 ± 10.87	37.87	78.50	

### Occupation ratio and fatty infiltration

**Figure 1:**
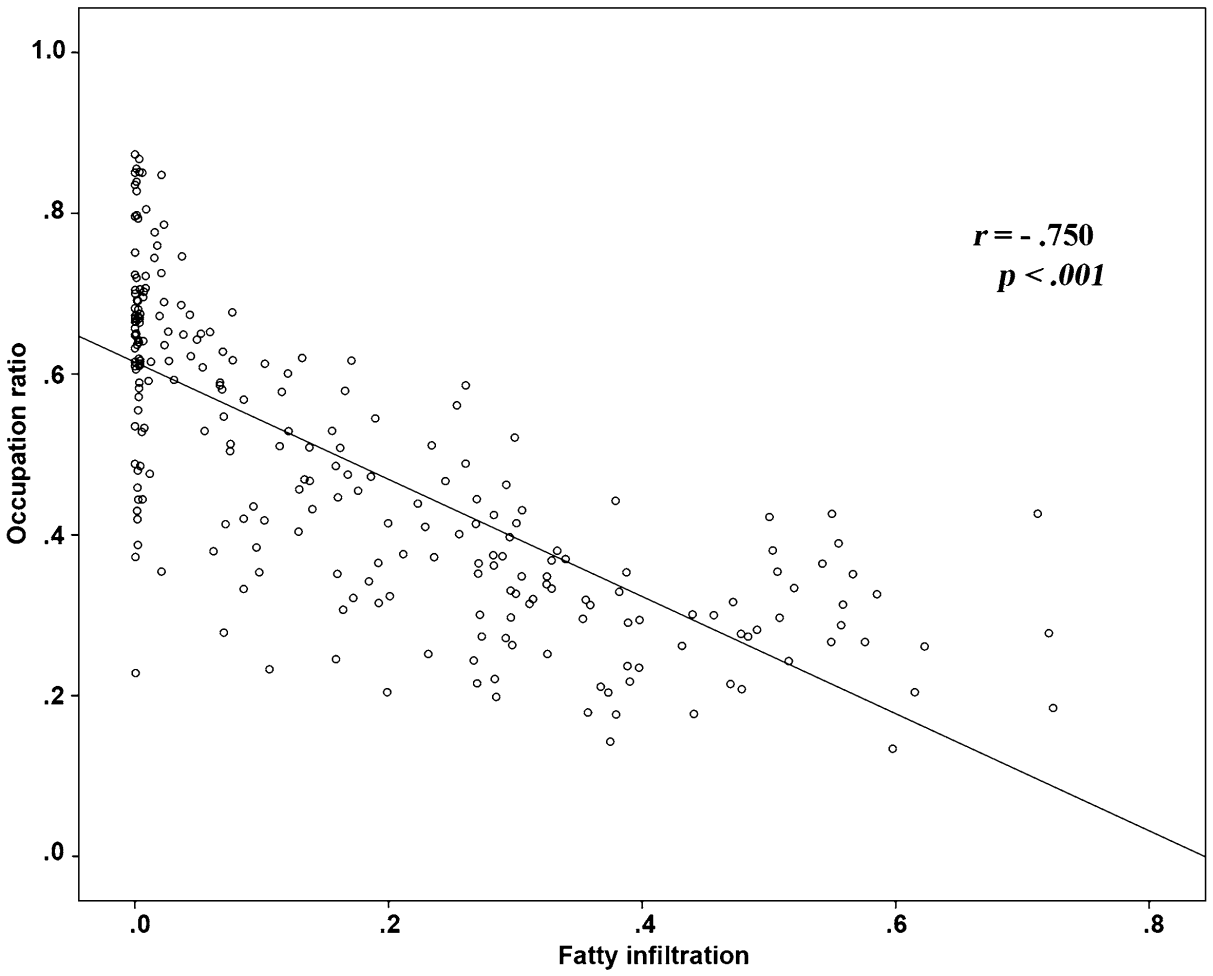
Correlation between the fatty infiltration of supraspinatus muscle using Otsu thresholding and occupation ratio of supraspinatus muscle based on segmentation with the CNN.

**Figure 2:**
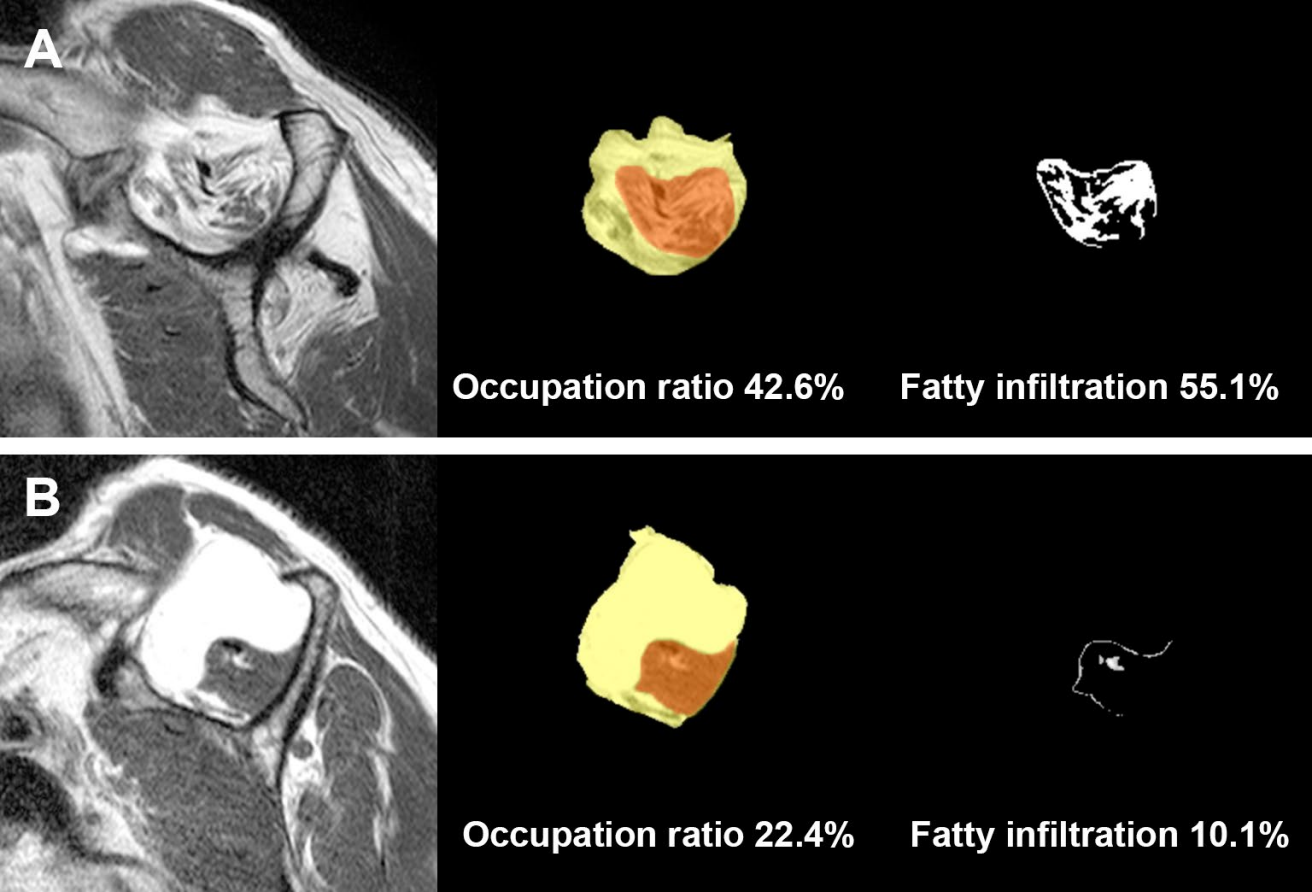
Outlier cases related to occupation ratio and fatty infiltration. Occupation ratio and fatty infiltration exhibited a strong negative correlation, which was statistically significant from the study. However, some cases reported relatively high occupation ratios and fatty infiltrations (**A**), while others reported relatively low values (**B**).

From the analysis of the correlation between the occupation ratio and fatty infiltration, the greater occupation ratio was strongly negatively correlated with fatty infiltration, and this correlation was statistically significant (r = − 0.750, *p* < 0.001) (Fig. 1). However, there were several outliers in the trends in the study. Some cases showed relatively high occupation ratios with high fatty infiltrations, while others showed relatively low occupation ratios with low fatty infiltrations (Fig. 2).

## Discussion

Rotator-cuff tears are the most frequent shoulder pathologies that cause pain and functional impairment^9,10^. Numerous authors have reported surgical methods and clinical outcomes of supraspinatus tendon repair^7,11–13^. Radiologic analysis of the rotator-cuff tendon has been used to predict the repairability of the supraspinatus tendon and likelihood of re-tear after arthroscopic repair^6,14^.

Atrophic changes and fatty infiltration of the rotator-cuff musculature are two of the more commonly accepted findings associated with large tears, and several methods for quantifying these changes have been described^12,14,15^. The scapular Y-view of the MRI, the lateral-most T1 sagittal MR image in which the scapular spine and body are in contact, is the base image for obtaining reliable indicators of the supraspinatus muscle status (for example, occupation ratio, tangent sign, and fatty infiltration)^14,16,17^. In general, these indicators are manually evaluated in the clinic. The occupation ratio is generally measured by tracing along the line of the outer edge of the supraspinatus muscle and inner margins of the supraspinatus fossa using a program cursor under the Picture Archiving and Communication System. This process is difficult and time-consuming, especially in cases where the margin of the supraspinatus muscle is irregular and rough^18^. Furthermore, fatty infiltration, measured using the Goutallier classification, has the limitation of being a subjective qualitative measurement. The relatively wide range of the five stages of the Goutallier classification has been cited as a potential reason for low reliability^19^, and some studies have shown only moderate or poor interobserver agreement^19–22^.

Recently, deep learning technology has been adopted to address many unsolved scientific and technical problems, and it has been applied in medical image analysis in recent studies^23,24^. In particular, CNNs have shown promise as high-capacity parametric models for image analysis by using a large number of parameters derived from training data^1,25,26^. Machine-learning-aided analysis can be trained with an enormous number of samples in a short time. An ideal system will have consistently accurate and precise diagnoses and would have the same diagnostic result given repeated input. The present study compared the abilities of humans and deep convolutional networks when detecting the region of segmentation of the supraspinatus muscle and supraspinatus fossa. As tears progress, muscles undergo retraction and fat infiltration related to atrophy^27^. In the context of cuff tears, the measurement of muscle atrophy, such as the occupation ratio of the supraspinatus muscle, has been considered an important prognostic indicator^28,29^. The CNN exhibited excellent agreement with the clinicians in both areas of segmentation. From the accurate segmentation, we can also easily obtain the occupation ratio, which is the proportion of the supraspinatus muscle from the supraspinatus fossa.

The assessment of fatty infiltration in the setting of rotator-cuff tears affects clinical decision-making, because the presence of fatty infiltration of 50% or more is a relative contraindication to rotator-cuff repair^30^. Thus, qualitative assessments of the supraspinatus muscle have been considered important for rotator-cuff tendon surgery and have been widely used in clinical studies on shoulder pathology^6,11,12^. In the present study, we proposed a modified Otsu thresholding technique to evaluate fatty infiltration in the supraspinatus muscle. Binarization algorithms include global fixed thresholding, locally adaptive thresholding, and hysteresis thresholding. The present study aims to detect optimal thresholds in a resgion of interest (ROI) where the visual structural characteristics change. Otsu thresholding is a global fixed thresholding methodthat has excellent performance. It is widely known and has been used in several previous studies on medical image analysis^31,32^. The result produced a binary image of nonparametric and unsupervised threshold selection data on a gray-level histogram. Thus, it enables detection of the fat portion from muscle without having to adjust the brightness to calculate the exact proportion of fatty infiltration. A previous study attempted to use quantitative MRI measurements of the fat fraction in rotator-cuff tendons and compared these with the Goutallier scores^33^. Increasing fat fraction correlated well with a higher Goutallier scores, aside from grades 3 (27.5%) and 4 (26.2%), for which there was no difference. Therefore, the authors recommend that the application of the model to the highest or lowest range should be interpreted with caution. Additionally, the authors used manual outlining of rotator-cuff muscle areas on each MRI slice, which was time-consuming and may have introduced methodological bias. In the present study, fatty infiltration by modified Otsu increased with higher Goutallier grades, and the differences between each grade were statistically significant. Grade 3 of the Goutallier classification, defined as equal amounts of fat and muscle, showed a mean of 42.86% fatty infiltration. Grades 2 and 4 reported 20.10% and 55.79% fatty infiltration, respectively.

Because the occupation ratio and fatty infiltration are related to disease severity, there have been many related studies^6,13,30^. Furthermore, as the severity of the rotator-cuff tear increases, atrophy and fatty infiltration of the supraspinatus muscle have been found to be more serious on MR images^4^. In the present study, there was a strong negative correlation between the occupation ratio via CNN and fatty infiltration via Otsu thresholding, which was also documented in the literature^11,12,27^. However, some cases showed a disparity between the occupation ratio and fatty infiltration. Fatty infiltration of the rotator cuff tendon tear is known to be a multifactorial process with proposed etiologies including chronicity, traction neuropathy, loss of muscle tension resulting in architectural changes, and physiological changes^4,33^. Because the fat portion inside the supraspinatus muscle and outside the muscle has similar signal intensity, it is critical to properly annotate the outline of the supraspinatus muscle. Using the software in the present study, we selected the scapular Y-view and annotated the supraspinatus muscle to simultaneously trace serial images. This helped us detect the outline of the tendon/muscle and distinguish the neurovascular structure, which had a similar signal intensity as tendons.

This study makes several valuable contributions to the literature. The most important advantage is that the analysis process is objective and saves time. Numerous MR image analyses of muscle atrophy and fatty infiltration can be performed in a shorter time, free from human errors. Another advantage is external validity. Our analysis was performed using a freeware computer program that can analyze and calculate muscle atrophy and fatty infiltration of scapular Y-view MR images in less than a second. Although it is not known what effects the sample bias/features and noise may have on external comparisons, we expect that this could be handled by modifying the algorithm. Lastly, the high performance of CNN for detecting muscle from MR images reveals the possibility of its application to other musculoskeletal areas.

Our study had some limitations. First, the number of original images was relatively small compared with other deep-learning studies. Second, clinical factors were not considered. Because the present study was an image analysis study, the observers were blinded to the clinical data. Based on the reliability of our analysis, clinical data should be evaluated to determine whether the image analysis is correlated with actual disease severity and whether it offers anything of clinical importance, as in previous studies^34,35^. Lastly, we used the data from a solitary MRI scanner. Although the process can have external validity, it is not known what sample bias/features and noise may have on external comparisons.

In summary, the proposed CNN showed fast and accurate segmentation of the supraspinatus muscle and fossa from shoulder MRI, which enabled us to automatically calculate the occupation ratio. Quantitative evaluation using the modified Otsu thresholding technique is a good method for calculating the proportion of fatty infiltration in the supraspinatus muscle. We expect that this can improve the efficiency and objectivity of diagnoses by quantifying the index used in shoulder MRI.

## Methods

This study was reviewed and approved by the Institutional Review Board (Institutional review board no.2019–05-109–001) of the Samsung Medical Center, and the requirement for informed consent was waived. Data collection and all experiments were performed in accordance with the Declaration of Helsinki.

### Patient selection

We randomly selected 250 patients who visited the outpatient clinic for shoulder MRI at the Samsung Medical Center between January 2015 and December 2016. All personal information was anonymized, and clinical data, including diagnoses, were ignored. A random number table was used for extraction. Only shoulder MR images were downloaded to evaluate atrophy and fatty infiltration of the supraspinatus muscle. Patients with previous implants in the ipsilateral shoulder were excluded from the study. For the application of the deep-learning algorithm, only 512 × 512-pixel MR images were used. Finally, the shoulder MRI data of 240 among the 250 patients were enrolled for analysis. MRI was performed with a 3.0-T imager (Gyroscan Intera Achieva; Philips Medical Systems, Best, the Netherlands) using a dedicated receive-only shoulder coil. Conventional two-dimensional MR images were obtained with fat-suppressed T1-weighted fast spin echo sequences in the axial and oblique coronal planes parallel to the long axis of the supraspinatus tendon, and the oblique sagittal plane perpendicular to the long axis of the supraspinatus tendon (repetition time/echo time, 560–754/8–10 ms; section thickness, 3 mm; field of view, 16 cm; acquisition matrix number, 320 × 256; echo train length, 5).

### Data collection and annotation

Data were collected from the MRI slice as input, and the ground truth was extracted as the output. A sagittal oblique plane view with a scapular Y-shaped view image slice of the MRI was used as the input, and the ground truth was annotated with two regions of the supraspinatus fossa and muscle in the image slice.

**Figure 3:**
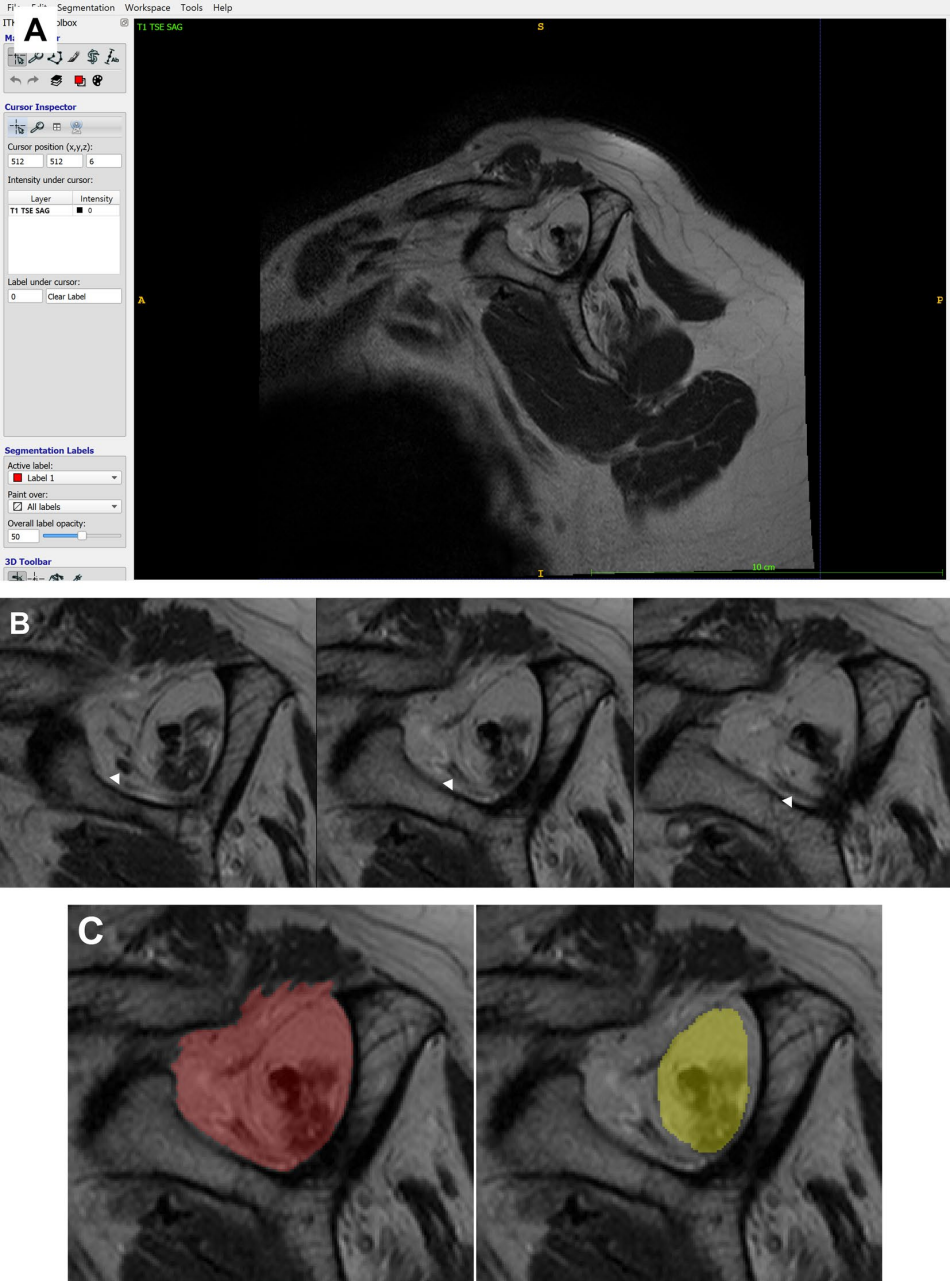
Annotation using ITK-SNAP software: (**A**) loading the MR image of oblique sagittal view on the ITK-SNAP; (**B**) detection of the scapular Y-view and identification of the margin of supraspinatus fossa and supraspinatus muscle. The arrowhead indicates the vessel around the supraspinatus muscle; (**C**) annotation of the supraspinatus fossa and supraspinatus muscle using the brush tool of ITK-SNAP.

To annotate the ground truth, we used ITK-SNAP, a freeware medical image labeling program^36^. The whole-series images of the T1-weighted sagittal oblique plane view were loaded onto the ITK-SNAP. The scapular Y view was identified, and an outline of the supraspinatus fossa and muscle was detected. The supraspinatus fossa and supraspinatus muscles were highlighted using a brush tool (Fig. 3).

The supraspinatus fossa and supraspinatus muscle were annotated according to a previous study^14^. First, in the shoulder MRI with a T1-weighted sagittal oblique view, we chose the most lateral image (i.e., the Y-shaped view) with the scapular spine in contact with the scapular body. Annotation of the supraspinatus fossa area was performed along the inner-bone margin of the Y-shaped scapula, inferior border of the trapezius, and inner-bone margin of the distal clavicle. When annotating the muscle area, the area drawn along the outer margin of the supraspinatus muscle in the supraspinatus fossa area was annotated as a margin, and the neurovascular structure outside the muscle area was excluded. In cases where it was difficult to accurately determine the neurovascular structure with similar signal intensities adjacent to the muscle in a segmentation, serial, anterior, and posterior images based on the segmented image were analyzed together to confirm the positions of vessels and nerves.

Fatty infiltration, measured via Goutallier grading, was performed with annotations of the supraspinatus muscle. According to this method, grade 0 denotes normal muscle tissue; grade 1, fatty streaking; grade 2, more muscle tissue than fat; grade 3, equal fat and muscle tissue; and grade 4, more fat than muscle^37^. All annotations and grading were performed by two orthopedic specialist surgeons at the shoulder and elbow clinic. Any disagreement between surgeons was discussed with a radiologist with expertise in musculoskeletal disease until a consensus was reached.

### Deep learning for segmentation using a CNN

**Figure 4:**
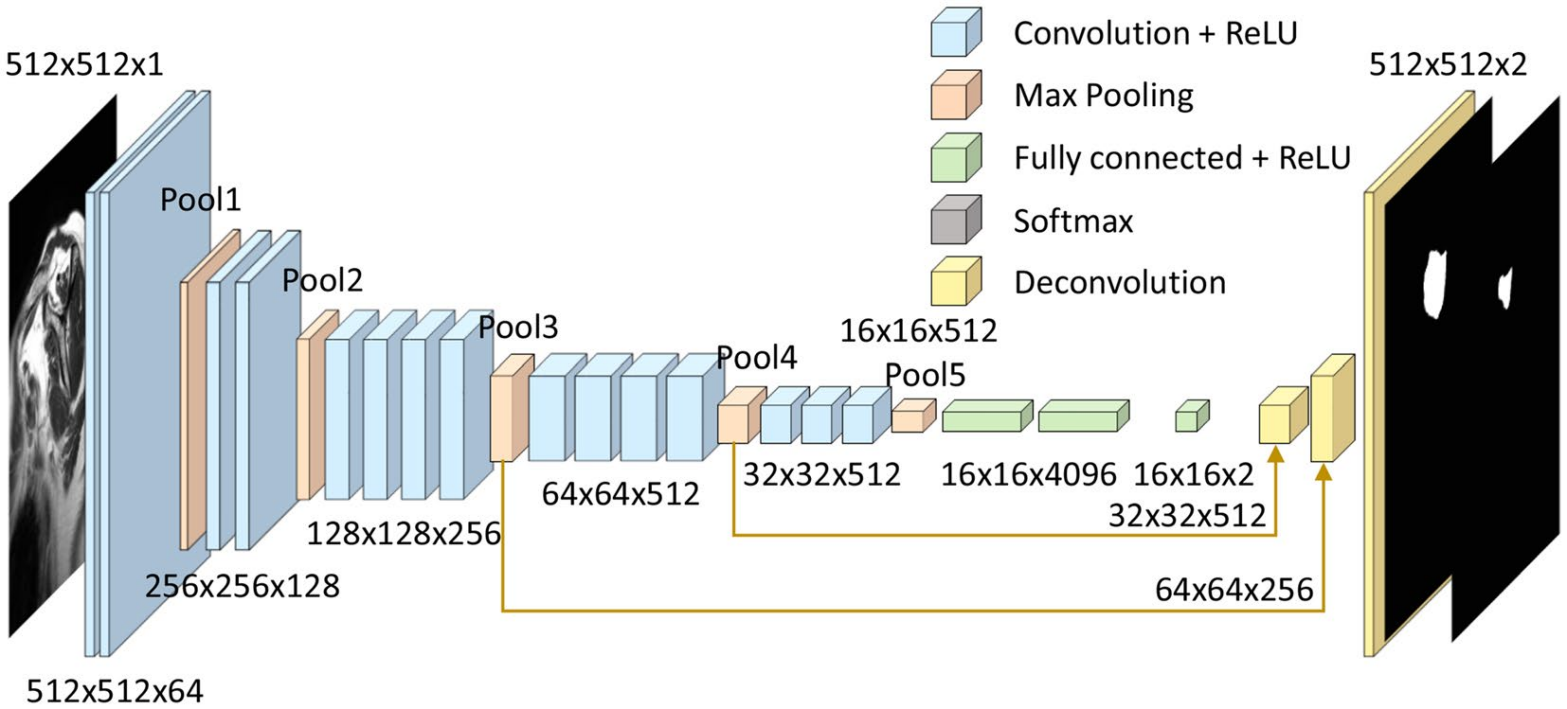
Schematic structure of the fully convolutional network used for algorithm development.

We used a CNN, where the detailed schematic structure comprised 15 convolution layers and five pooling layers based on the VGG19 network^38^. Three fully convolutional layers were added for semantic segmentation (Fig. 4). The feature maps of the 3rd and 4^th^ pooling layers were used to obtain the output via the deconvolution and up-sample processes at the end of the network. The prediction was defined as the output image after deep learning using the ground truth.

### Data augmentation

Data augmentation is necessary to mitigate the lack of data common in developing algorithms for medical imaging using deep learning; this study used flip and brightness controls^39^. The flip algorithm flips the MRI slices left and right, and at this stage, the amount of data is doubled. With these techniques, we obtained additional training data and resolved the imbalance problems associated with the number of right and left shoulders. Augmentation using brightness was applied based on histogram analysis^40^. The brightness of the entire data was analyzed and classified into five stages, and the data were augmented by applying the histogram matching technique of the input image to the average of the histogram distribution within each brightness stage^41^. At this stage, the amount of data increased by a factor of five. After augmentation, two augmentations were superimposed to increase the amount of data ten times.

The augmentation method described above was applied only to the training course. In the k-fold cross-validation process, the validation set was initially separated and fixed as the original image, and learning was performed by applying augmentation when learning the remaining training sets, excluding the validation set. Therefore, the training and validation images were completely different images of the patients.

### k-fold cross-validation

Ten-fold cross-validation, which was used in a previous study, was performed to evaluate the performance of the developed algorithm^42^. Because the total number of images used for training in the network was 240 and the k value was set to 10, 24 images were used as the validation set and 216 images as the training set. The validation set images were randomly selected 10 times but were not duplicated, and the remaining images were used for training each time. Models using 10 different validation sets were trained, and the parameters of each model were distinct. Then, the performance average of the 10 models was used as the result. These results were used to derive the area division images to analyze the area results. To ensure reliability, augmentation was performed only on the training dataset after the entire dataset was divided into k segments to prevent similar inputs between the training and test sets.

### Adaptive Otsu thresholding

After segmentation of the supraspinatus muscle, fatty infiltration of the muscle substance was evaluated using Otsu thresholding, which is characterized by its nonparametric and unsupervised nature of threshold selection on a gray-level histogram^43^. The output of the Otsu thresholding technique is a binary image. Thus, it has been applied to several medical image studies to detect outlines of organs and lesions and distinguish them from the background^2,44,45^. If the intensity distribution in the image is clear, a more accurate classification is possible. Therefore, Otsu thresholding is expected to easily detect a threshold value at the pixel intensity, which maximizes the differences between the foreground (bright) and background (dark) pixels^15^.

**Figure 5:**
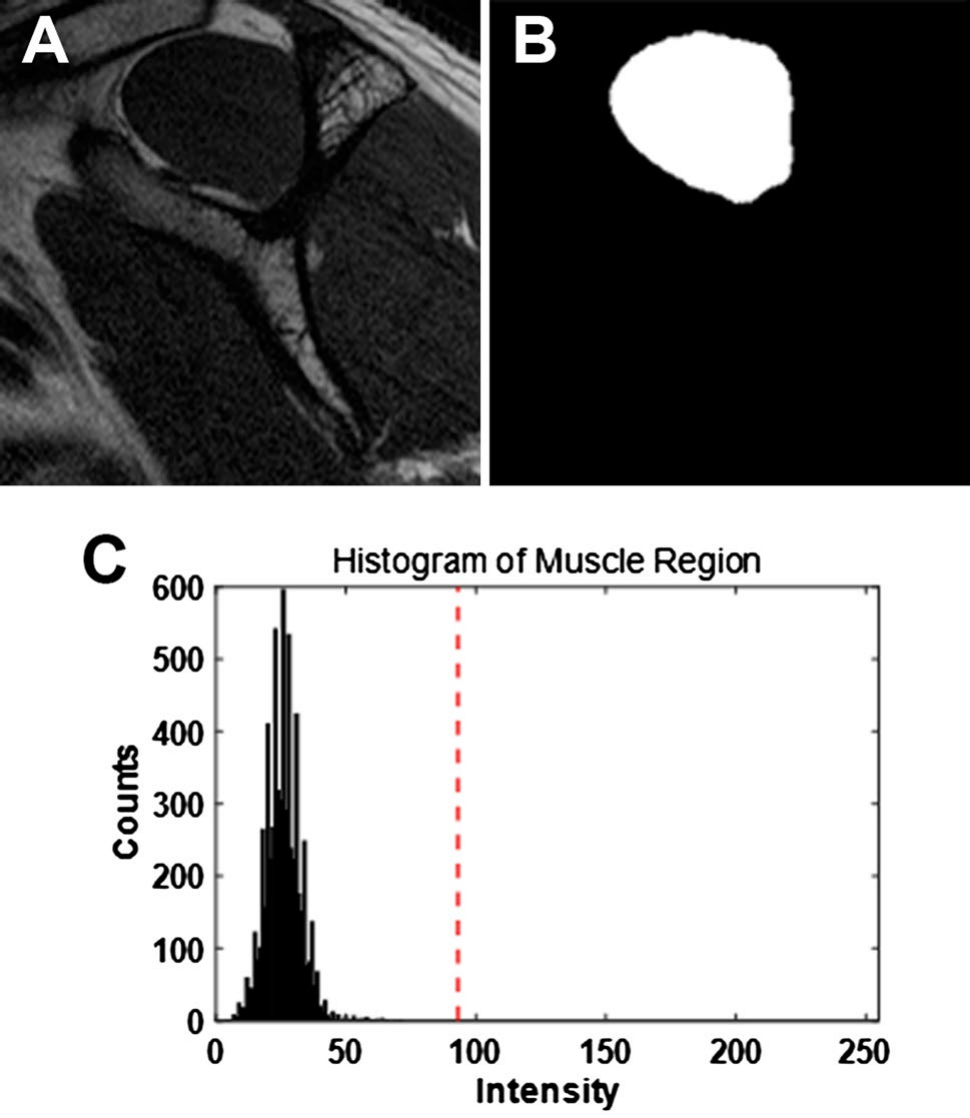
Analysis result of image without fatty infiltration in the muscle region. (**A**) Original image. (**B**) ROI used for histogram analysis. (**C**) Histogram graph in ROI. The red dotted line is a threshold value (intensity level = 93) automatically determined by Otsu thresholding. The maximum value of the intensity detected in the ROI is 71;therefore, the counts value exceeding 71 intensity level is 0.

**Figure 6:**
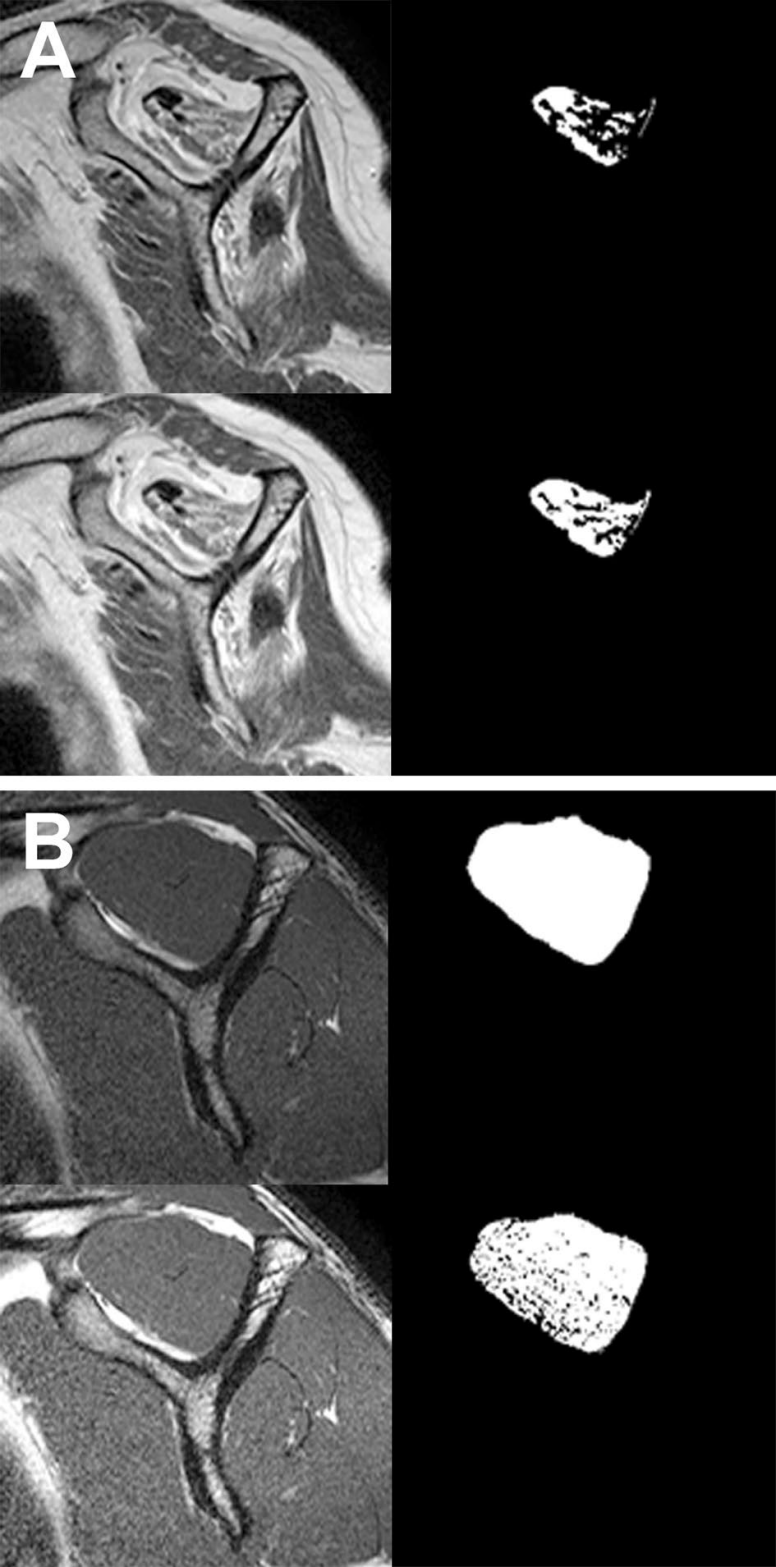
Comparison of the muscle region extracted by applying Otsu thresholding to the original image (row 1) and after image adjustment (row 2). (**A**) An example of a case where image adjustment improves the performance of Otsu thresholding because the severity of the Goutallier grade is high. (**B**) Example of when it is better not to adjust the image because the severity of the Goutallier grade is low and there is little fatty infiltration in the muscle area.

**Figure 7:**
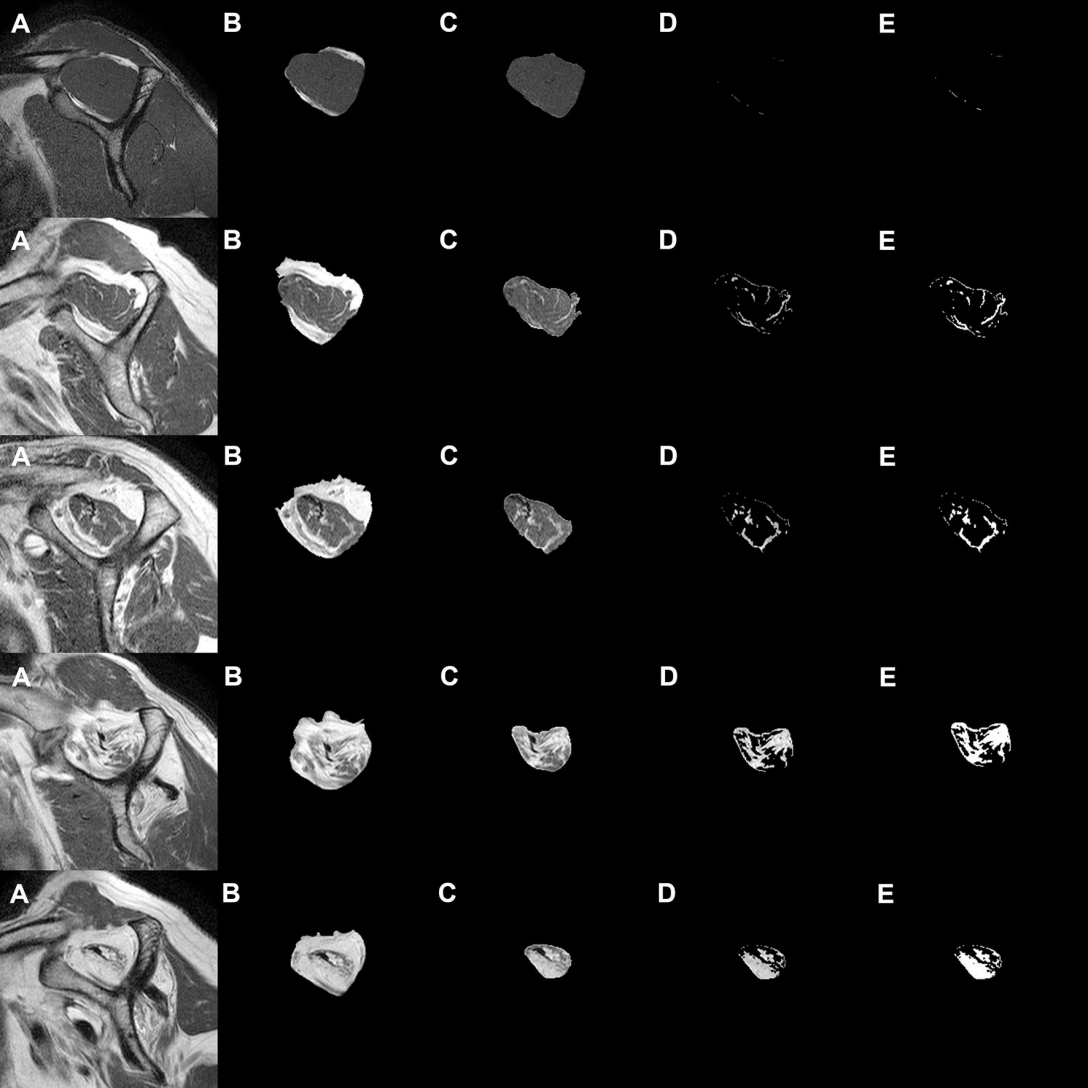
Image analysis steps of the proposed method for supraspinatus muscle segmentation by CNN and the fatty infiltration analysis by Otsu thresholding: (**A**) original MR image; (**B**) supraspinatus fossa segmentation by CNN; (**C**) supraspinatus muscle segmentation by CNN; (**D**) Otsu thresholding fatty infiltration image from the area of supraspinatus muscle; and (**E**) post processed final binary image. This includes black (supraspinatus muscle) and white (fatty infiltration) conversions of Otsu thresholding fatty infiltration image.

Muscle and fatty infiltration within the detected muscle area were significantly distinguished in the ROI using Otsu thresholding. Additionally, the threshold for detecting only the muscle region was determined, even in the absence of fatty infiltration in the muscle (Fig. 5). However, in patients with high severity, the tissue is not uniform because of internal degeneration; therefore, even if it was the same fat tissue, the performance was not good because the boundary of the tissue was not clear; for example, the intensity was expressed in several stages. To address this, we attempted to adjust the image using histogram equalization and better performance was obtained for patients with high severity after equalization (Fig. 6A). However, if the low-intensity area of the input image is large, simply using histogram equalization reduces the dynamic range and causes a data wash-out problem^46^ (Fig. 6B). This problem occurs in patients with low severity, that is, those with low fatty infiltration. Therefore, to improve and to stabilize the performance according to severity, the application of histogram equalization was determined through a statistical analysis of the intensity in the muscle region. Because the intensity distribution in the ROI changes with fatty infiltration, the standard deviation of intensity increases with severity. Based on the Goutallier grade diagnosed in advance by the clinician, the standard deviation of image brightness in the ROI was 28.47, on average, for grade 2, and the upper limit was approximately 35.95. The average score for grade 3 was 35.18. When the standard deviation of the ROI was 35 or more, histogram equalization was applied within the muscle region before applying the Otsu threshold. The precise values were determined empirically, thus allowing improvement of detection results for fatty infiltration regions in the images of all subjects, regardless of the severity. The final product of Otsu thresholding in this study showed white pixels representing the fatty parts and black pixels representing the muscle parts, following the supraspinatus muscle segmentation (Fig. 7).

### Evaluation metrics

We evaluated the performance of our models in terms of the overlap between the ground truth human measures and segmentation results from our models. The DSC, defined as the ratio of the overlap to the mean area of two segmentations, was used as the main evaluation metric:

1 $${\text{DSC}} = ~\frac{{2*{\text{Area}}_{{{\text{overlapped}}}} }}{{{\text{Area}}_{{{\text{ground}}\;{\text{truth}}}} + {\text{Area}}_{{{\text{prediction}}}} }}$$

DSC was evaluated to compare the similarities using an index ranging between 0 (no segmentation overlap) and 1 (perfect segmentation overlap)^47^. Although the absolute value of DSC is difficult to interpret, some previous studies proposed that > 0.70 indicates excellent agreement between measurement pairs^42,48^. Accuracy, sensitivity, and specificity were used to evaluate the ability of the models to detect the regions. The RAD was calculated to determine the extent to which the size of the segmented area was underestimated or overestimated from the ground truth^49^:

2 $${\text{RAD}} = \frac{{|{\text{Area}}_{{{\text{ground~}}\;{\text{truth}}}} \left| { - {\text{~}}} \right|{\text{Area}}_{{{\text{prediction}}}} |}}{{|{\text{Area}}_{{{\text{ground}}\;{\text{~truth}}}} |}} \times 100$$

Based on the annotated ground truth, we also calculated the occupation ratio as the area of the supraspinatus muscle over the area of the supraspinatus fossa, which is one of the methods used to evaluate muscle atrophy^14^.

From the annotated ground truth, fat and non-fat components were classified using the Otsu thresholding process. The proportion of fatty pixels inside the muscle area was calculated as a quantitative measure of fatty infiltration, usually between 0, indicating no fat in the supraspinatus muscle, and 1, indicating 100% fatty infiltration.

### Statistical analysis

The intra-observer and inter-observer reliabilities of each measurement were determined by calculating the weighted κ index values or intraclass correlation coefficient. Comparison of fatty infiltration using Otsu thresholding on each Goutallier grade was performed using one-way analysis of variance, followed by Bonferroni post hoc analysis. Correlation analysis using the Pearson correlation coefficient helped to identify the relationship between occupation ratio and fatty infiltration. Statistical analysis was performed using R statistical software Version 3.4.0 (the metaphor package: a Meta-Analysis Package for R; R Foundation for Statistical Computing, Vienna, Austria) and the Statistical Package for the Social Sciences (SPSS) software package (version 20.0; SPSS, Chicago, IL, USA). The level of significance was set at *p* < 0.05.

## Supplementary Information

Supplementary Information 1.

Supplementary Information 2.

## References

[1] Chung SW (2018). Automated detection and classification of the proximal humerus fracture by using deep learning algorithm. Acta Orthop..

[2] Thakran S, Chatterjee S, Singhal M, Gupta RK, Singh A (2018). Automatic outer and inner breast tissue segmentation using multi-parametric MRI images of breast tumor patients. PLoS ONE.

[3] Prasoon, A. *et al.* in *International conference on medical image computing and computer-assisted intervention.* 246–253 (Springer).

[4] Nakagaki K, Ozaki J, Tomita Y, Tamai S (1994). Alterations in the supraspinatus muscle belly with rotator cuff tearing: evaluation with magnetic resonance imaging. J. Shoulder Elbow Surg..

[5] Chung SW (2013). Is the supraspinatus muscle atrophy truly irreversible after surgical repair of rotator cuff tears?. Clin. Orthop. Surg..

[6] Jeong HY, Kim HJ, Jeon YS, Rhee YG (2018). Factors predictive of healing in large rotator cuff tears: Is it possible to predict retear preoperatively?. Am. J. Sports Med..

[7] Godenèche A (2017). Fatty infiltration of stage 1 or higher significantly compromises long-term healing of supraspinatus repairs. J. Shoulder Elbow Surg..

[8] Somerson JS, Hsu JE, Gorbaty JD, Gee AO (2016). Classifications in brief: goutallier classification of fatty infiltration of the rotator cuff musculature. Clin. Orthop. Relat. Res..

[9] Ro KH (2015). Status of the contralateral rotator cuff in patients undergoing rotator cuff repair. Am. J. Sports Med..

[10] Karasuyama M (2020). Clinical results of conservative management in patients with full-thickness rotator cuff tear: a meta-analysis. Clin Shoulder Elbow.

[11] Park YB, Ryu HY, Hong JH, Ko YH, Yoo JC (2016). Reversibility of supraspinatus muscle atrophy in tendon-bone healing after arthroscopic rotator cuff repair. Am. J. Sports Med..

[12] Kim HB, Yoo JC, Jeong JY (2019). Evaluation of muscular atrophy and fatty infiltration using time-zero magnetic resonance imaging as baseline data, after rotator cuff repair. Clin. Shoulder Elbow.

[13] Hamano N (2017). Does successful rotator cuff repair improve muscle atrophy and fatty infiltration of the rotator cuff? A retrospective magnetic resonance imaging study performed shortly after surgery as a reference. J. Shoulder Elbow Surg..

[14] Jeong JY, Chung PK, Lee SM, Yoo JC (2017). Supraspinatus muscle occupation ratio predicts rotator cuff reparability. J. Shoulder Elbow Surg..

[15] Lee D (2020). Threshold-based quantification of fatty degeneration in the supraspinatus muscle on MRI as an alternative method to Goutallier classification and single-voxel MR spectroscopy. BMC Musculoskelet. Disord..

[16] Thomazeau H, Rolland Y, Lucas C, Duval J-M, Langlais FJAOS (1996). Atrophy of the supraspinatus belly assessment by MRI in 55 patients with rotator cuff pathology. Acta Orthop. Scand..

[17] Zanetti, M., Gerber, C. & Hodler, J.J.I.r. Quantitative assessment of the muscles of the rotator cuff with magnetic resonance imaging. **33,** 163–170 (1998). 10.1097/00004424-199803000-00006 9525755

[18] Tae S-K (2011). Evaluation of fatty degeneration of the supraspinatus muscle using a new measuring tool and its correlation between multidetector computed tomography and magnetic resonance imaging. Am. J. Sports Med..

[19] Spencer EE (2008). Interobserver agreement in the classification of rotator cuff tears using magnetic resonance imaging. Am. J. Sports Med..

[20] Lesage P (2002). Reproducibility of CT scan evaluation of muscular fatty degeneration Intra-and interobserver analysis of 56 shoulders presenting with a ruptured rotator cuff muscles. Rev. Chir. Orthop. Reparatrice Appar. Mot..

[21] Oh JH, Kim SH, Choi J-A, Kim Y, Oh CH (2010). Reliability of the grading system for fatty degeneration of rotator cuff muscles. Clin. Orthopaed. Relat. Res..

[22] Slabaugh MA (2012). Interobserver and intraobserver reliability of the Goutallier classification using magnetic resonance imaging: proposal of a simplified classification system to increase reliability. Am. J. Sports Med..

[23] Wang G, Han Y (2021). Convolutional neural network for automatically segmenting magnetic resonance images of the shoulder joint. Comput. Methods Prog. Biomed..

[24] Shim E (2020). Automated rotator cuff tear classification using 3D convolutional neural network. Sci. Rep..

[25] LeCun Y, Bengio Y, Hinton G (2015). Deep learning. Nature.

[26] Krizhevsky, A., Sutskever, I. & Hinton, G.E. in *Advances in neural information processing systems.* 1097–1105.

[27] Nakagaki K, Ozaki J, Tomita Y, Tamai S (1996). Fatty degeneration in the supraspinatus muscle after rotator cuff tear. J. Shoulder Elbow Surg..

[28] Goutallier D, Postel J-M, Gleyze P, Leguilloux P, Van Driessche S (2003). Influence of cuff muscle fatty degeneration on anatomic and functional outcomes after simple suture of full-thickness tears. J. Shoulder Elbow Surg..

[29] Harryman D (1991). Repairs of the rotator cuff Correlation of functional results with. J. Bone Joint Surg. Am..

[30] Ashry R (2007). Muscle atrophy as a consequence of rotator cuff tears: should we compare the muscles of the rotator cuff with those of the deltoid?. Skeletal Radiol..

[31] Feng Y, Zhao H, Li X, Zhang X, Li H (2017). A multi-scale 3D Otsu thresholding algorithm for medical image segmentation. Digital Signal Process..

[32] Merzban MH, Elbayoumi M (2019). Efficient solution of Otsu multilevel image thresholding: a comparative study. Expert Syst. Appl..

[33] Lee S (2015). Magnetic resonance rotator cuff fat fraction and its relationship with tendon tear severity and subject characteristics. J. Shoulder Elbow Surg..

[34] Seitz AL (2017). Quantifying variation in intramuscular fat infiltration in patients with rotator cuff tears. J. Shoulder Elbow Surgery.

[35] Hansen LM (2021). Clinical Y-view versus 3-dimensional assessments of intramuscular fat in patients with full-thickness rotator cuff tears. Clin. Imaging.

[36] Yushkevich PA (2006). User-guided 3D active contour segmentation of anatomical structures: significantly improved efficiency and reliability. Neuroimage.

[37] Fuchs B, Weishaupt D, Zanetti M, Hodler J, Gerber C (1999). Fatty degeneration of the muscles of the rotator cuff: assessment by computed tomography versus magnetic resonance imaging. J. Shoulder Elbow Surg..

[38] Simonyan, K. & Zisserman, A. Very deep convolutional networks for large-scale image recognition. arXiv preprint arXiv:1409.1556v6 (2014).

[39] Perez, L. & Wang, J. The effectiveness of data augmentation in image classification using deep learning. arXiv preprint arXiv:1712.04621v1arXiv:1712.04621v1 (2017).

[40] Shorten, C. & Khoshgoftaar, T.M.J.J.o.B.D. A survey on image data augmentation for deep learning. **6,** 1–48 (2019). 10.1186/s40537-021-00492-0 PMC8287113 34306963

[41] Ma, J.J.a.p.a. Histogram Matching Augmentation for Domain Adaptation with Application to Multi-Centre, Multi-Vendor and Multi-Disease Cardiac Image Segmentation. (2020).

[42] Kim JY (2019). Development of an automatic muscle atrophy measuring algorithm to calculate the ratio of supraspinatus in supraspinous fossa using deep learning. Comput. Methods Prog. Biomed..

[43] Otsu N (1979). A threshold selection method from gray-level histograms. IEEE Trans. Syst. Man Cybern..

[44] BahadarKhan K, Khaliq AA, Shahid M (2016). A morphological hessian based approach for retinal blood vessels segmentation and denoising using region based otsu thresholding. PLoS ONE.

[45] Kaur R (2017). Thresholding methods for lesion segmentation of basal cell carcinoma in dermoscopy images. Skin Res. Technol..

[46] Toet A, Wu TJPR (2015). Infrared contrast enhancement through log-power histogram modification. J. Pattern Recogn. Res..

[47] Taghizadeh, E. *et al.* Deep learning for the rapid automatic quantification and characterization of rotator cuff muscle degeneration from shoulder CT datasets. 1–10 (2020). 10.1007/s00330-020-07070-7 PMC7755645 32696257

[48] Zijdenbos AP, Dawant BM, Margolin RA, Palmer AC (1994). Morphometric analysis of white matter lesions in MR images: method and validation. IEEE Trans. Med. Imag..

[49] Lee H, Kang KE, Chung H, Kim HC (2018). Automated segmentation of lesions including subretinal hyperreflective material in neovascular age-related macular degeneration. Am. J. Ophthalmol..

