# Supplementary material for: Deep-learning framework and computer assisted fatty infiltration analysis for the supraspinatus muscle in MRI
Source: Sci Rep. 2021 Jul 23;11:15065. doi: 10.1038/s41598-021-93026-w (PMC8302634; doi:10.1038/s41598-021-93026-w)
Supplement: Supplementary file 1 — Supplementary Information 1. [file 41598_2021_93026_MOESM1_ESM.pdf]

NOTIFICATION LETTER

OF SMC IRB REVIEW SUMMARY

Principal Investigator :	Jae-Chul Yoo, M.D. PhD.<br>Professor, Department of Orthopedic Surgery, SMC
SMC IRB File No. :	2019-05-109-001
Protocol Title :	A Deep Learning Framework and Computer Assisted Analaysis for Supraspinatus Muscle in MRI

☑ **Approval** ☐ Contingent approval

* Please append details or summarize reason(s) for determination below:
SMC IRB has confirmed that the investigator agreed with our recommendations for protocol alterations and the alterations were reflected/responded to the IRB accordingly. It presents less than minimal risk to the research subject and meets the requirements for expedited review.

1. Research protocol changed
   - IRB recommendations reflected
2. Target subject number matched (200 -> 240)
3. Researchers added & their GCP training records confirmed
   - Heeseol Park, Baek Hwan Cho

SMC IRB has approved the conduct of the research and determined one-year interval for continuing review of this research. To continue the research after the expiration of IRB approval, the PI must submit “Research Progress Report (www.e-IRB.com ->Log-in -> e-IRB Main Bar -> Download Forms -> Reference Room)” for the continuing review after Aug. 20, 2020.

<u>Date of Review : Oct. 21, 2019</u>

Institutional Review Board
Samsung Medical Center
#81, Irwon-Ro, Gangnam-Gu, Seoul, Korea, 135-710

APPROVED
SAMSUNG
삼성서울병원
Institutional Review Board

CONTENTS OF IRB REVIEW

Principal Investigator :	Jae-Chul Yoo, M.D. PhD.<br>Professor, Department of Orthopedic Surgery, SMC
SMC IRB File No. :	2019-05-109-001
Protocol Title :	A Deep Learning Framework and Computer Assisted Analaysis for Supraspinatus Muscle in MRI

DOCUMENT REVIEWED

- Research protocol

Issued by

Seil Jang
IRB Secretary for Administration
Samsung Medical Center

Nov. 08, 2019
Date

APPROVED SAMSUNG 삼성서울병원 Institutional Review Board

IRB Information

Institution :	Samsung Medical Center (SMC)
Address :	#81 Irwon-ro, Gangnam-gu, Seoul, Korea
IRB President :	Prof. Suk-koo Lee, M.D., Ph.D.<br>Dept. of Pediatric Surgery

SMC IRB abides by the guideline of ICH and GCP.

SMC IRB and the Institution are registered at Office for Human Research Protections (OHRP), U.S. Dept. of Health & Human Services, U.S.A..

Related SMC IRB registration and accreditation information is as follows.

＊ Identifier No. (U.S. OHRP)

Organization :	IORG0000545 - Samsung Med Ctr
Assurance :	FWA00002750
IRBs :	IRB #1 : IRB00000877
	IRB #2 : IRB00005489
	IRB #3 : IRB00005490
	IRB #4 : IRB00005491
	IRB #5 : IRB00006642
	IRB #6 : IRB00006643
	IRB #7 : IRB00008480
	IRB #8 : IRB00008481
	IRB #9 : IRB00011389
	IRB#10 : IRB00011390

＊ Samsung Medical Center has earned the accreditation from the Association for the Accreditation of Human Research Protection Programs, Inc. (AAHRPP) on June 16, 2006.

This research protocol is reviewed by expedited IRB review process

APPROVED
SAMSUNG
삼성서울병원
Institutional Review Board
