# Supplementary material for: Deep-learning framework and computer assisted fatty infiltration analysis for the supraspinatus muscle in MRI
Source: Sci Rep. 2021 Jul 23;11:15065. doi: 10.1038/s41598-021-93026-w (PMC8302634; doi:10.1038/s41598-021-93026-w)
Supplement: Supplementary file 2 — Supplementary Information 2. [file 41598_2021_93026_MOESM2_ESM.docx]

**Post-Hoc analysis for Fatty infiltration by Otsu Thresholding**

| **Variable** | | **Fatty_infiltration_** | | **p-value*** | **Bonferroni Post-Hoc analysis** | | | | | | | | | | | | | | | | | | | |
| --- | --- | --- | --- | --- | --- | --- | --- | --- | --- | --- | --- | --- | --- | --- | --- | --- | --- | --- | --- | --- | --- | --- | --- | --- |
|  |  | **N** | **Mean ± SD** |  | **Grade 0 vs Grade 1** | | **Grade 0 vs Grade 2** | | **Grade 0 vs Grade 3** | | **Grade 0 vs Grade 4** | **Grade 1 vs Grade 2** | | **Grade 1 vs Grade 3** | | **Grade 1 vs Grade 4** | | | **Grade 2 vs Grade 3** | | **Grade 2 vs Grade 4** | | **Grade 3 vs Grade 4** | |
| Goutallier | Grade 0^a^ | 55 | 0.06 ± 0.14 | <.0001 | 0.0170 | <.0001 | | <.0001 | | <.0001 | | <.0001 | <.0001 | | <.0001 | | <.0001 | <.0001 | | <.0001 | | <.0001 | | <.0001 |
|  | Grade 1^b^ | 75 | 4.68 ± 7.21 |  |  |  |  |  |  |  |  |  |  |  |  |  |  |  |  |  |  |  |  |  |
|  | Grade 2^c^ | 68 | 20.10 ± 10.57 |  |  |  |  |  |  |  |  |  |  |  |  |  |  |  |  |  |  |  |  |  |
|  | Grade 3^d^ | 23 | 42.86 ± 10.41 |  |  |  |  |  |  |  |  |  |  |  |  |  |  |  |  |  |  |  |  |  |
|  | Grade 4^e^ | 19 | 55.79 ± 10.87 |  |  |  |  |  |  |  |  |  |  |  |  |  |  |  |  |  |  |  |  |  |

Note : The different alphabet letters have significant differences through the Bonferroni post-hoc analysis.

*one-way ANOVA

SD = standard deviation
